# Restoring Synaptic Balance in Schizophrenia: Insights From a Thalamo-Cortical Conductance-Based Model

**DOI:** 10.1093/schbul/sbaf149

**Published:** 2025-09-11

**Authors:** Lioba C S Berndt, Krish D Singh, Alexander D Shaw

**Affiliations:** Department of Psychology, Faculty of Health & Life Sciences, University of Exeter, Exeter, EX4 4QG, United Kingdom; Cardiff University Brain Research Imaging Centre, School of Psychology, Cardiff University, Cardiff, CF24 4HQ, United Kingdom; Department of Psychology, Faculty of Health & Life Sciences, University of Exeter, Exeter, EX4 4QG, United Kingdom

**Keywords:** computational psychiatry, DCM, mechanisms, NMDA dysconnectivity, GABA

## Abstract

**Background and Hypothesis:**

The dysconnectivity hypothesis of schizophrenia suggests that atypical neural communication underlies the disorder’s diverse symptoms. Building on this framework, we propose that specific synaptic disturbances within thalamo-cortical circuits contribute to an imbalance in excitation and inhibition, leading to alteration in oscillations. Our study investigates these alterations and explores whether synaptic restoration can remediate neural activity of schizophrenia and align it with healthy patterns.

**Study Design:**

We analyzed magnetoencephalography data from schizophrenia patients and healthy controls using dynamic causal modeling to identify synaptic differences in thalamo-cortical circuits. The analysis focused on N-methyl-D-aspartate (NMDA), α-amino-3-hydroxy-5-methyl-4-isoxazolepropionic acid (AMPA), gamma-aminobutyric acid type A (GABA-A), and gamma-aminobutyric acid type B (GABA-B) receptor-mediated connections. In silico synaptic restoration analysis simulated the effects of targeted adjustments to these receptor-mediated connections to assess whether altered neural activity in schizophrenia could be restored to match control patterns.

**Study Results:**

Schizophrenia patients showed statistically significant differences in increased NMDA receptor excitation in superficial pyramidal neurons and reduced GABA-B receptor inhibition between interneurons and pyramidal cells. Parameter recovery analysis revealed limitations for these specific parameters, suggesting that receptor-level interpretations should be made with caution. The in silico synaptic restoration analysis indicated that coordinated modifications across multiple synaptic pathways could potentially remediate neural activity to resemble healthy controls.

**Conclusions:**

This restoration approach suggests the complex nature of synaptic dysfunction in schizophrenia may involve coordinated changes across multiple synaptic parameters rather than isolated alterations. While our findings provide preliminary evidence extending the dysconnectivity theory of schizophrenia, the parameter recovery limitations suggest that specific receptor claims should be interpreted with caution.

## 1 Introduction

Schizophrenia is a severe mental disorder characterized by hallucinations, delusions, disorganized thinking, and impaired cognition.^1^ Despite extensive research, the precise neurobiological mechanisms underlying schizophrenia remain elusive. Understanding its pathology requires techniques for the in vivo study of neurobiological mechanisms and cortical circuitry. Traditional methods, such as post-mortem tissue analysis^2^^,^^3^ or non-invasive imaging,^4^ provide insights but have limitations in assessing inter-population connectivity and receptor dynamics.

Computational models of neuronal circuitry, such as dynamic causal models, offer a solution to bridge this micro-macro impasse. These models estimate synaptic processes and receptor dynamics from non-invasive magnetoencephalography/electroencephalography (M/EEG) data.^5^ Parameterized generative models based on biophysical equations, like the Hodgkin-Huxley model,^6^ can be inverted on empirical data features, including power spectral densities or event-related responses. This inversion estimates parameters reflecting connectivity strengths and receptor dynamics. Computational modeling enables inferences about brain dynamics at the spatial scale where drug mechanisms and neuropathological hypotheses of schizophrenia operate.^5^ This allows testing hypotheses about impaired receptor systems or connectivity and identifying pharmacological targets.^7^

Among prominent neurobiological models of schizophrenia, the *dysconnectivity hypothesis* has gained attention.^8^ It proposes that schizophrenia is a disorder of altered connectivity within and between brain networks. This hypothesis synthesizes earlier models, such as the dopamine hypothesis,^9^ focusing on dopaminergic dysregulation, and the glutamate hypothesis, implicating NMDA receptor hypofunction in cognitive and negative symptoms.^10^ The dysconnectivity hypothesis highlights that schizophrenia stems from disrupted coordination of brain regions rather than isolated neurotransmitter dysfunction.^8^ This manifests in cortico-cortical alterations and cortico-subcortical disruptions, particularly involving the thalamus, striatum, and hippocampus.^11–13^

Schizophrenia is also associated with disturbances in neural oscillations, particularly within the gamma-band (30-80 Hz) range.^14–17^ Reduced gamma power and altered peak frequencies are consistently observed in resting-state, aligning with the excitation-inhibition imbalance hypothesis.^18^ Disruptions between excitatory glutamatergic and inhibitory GABAergic systems underlie this imbalance. GABAergic dysfunction impairs inhibitory control, while the interplay between GABAergic inhibition and NMDA receptor-mediated excitation stabilizes thalamo-cortical circuits. Dysfunctions in these systems contribute to network-level disruptions observed in schizophrenia.^19^^,^^20^

The present study builds on findings by Shaw et al.,^15^ who investigated visual processing in schizophrenia using MEG. They demonstrated that individuals with schizophrenia showed reduced gamma frequency and impaired orientation discrimination performance. Using dynamic causal modeling, they identified reduced local synaptic connections in schizophrenia, with local inhibition correlating negatively with symptom severity. Effective connectivity between inhibitory interneurons and superficial pyramidal cells predicted orientation discrimination performance, underscoring the role of inhibitory circuits in perceptual processing. However, their convolution-based canonical microcircuit model^21^ lacked specificity in determining which receptor systems underpinned these differences. Since GABAergic and NMDA receptor systems are key to schizophrenia pathophysiology,^19^^,^^20^ understanding these systems is vital for identifying therapeutic targets.

To address these limitations, we applied a thalamocortical model^22^ explicitly incorporating receptor systems and thalamo-cortical interactions. We focused on the thalamus given its implication in schizophrenia pathophysiology, where thalamic volume reductions and disrupted connectivity are hallmarks^23^^,^^24^ and its role in visual gamma generation.^25^^,^^26^ This model provides deeper insights into mechanisms underlying observed oscillatory differences in schizophrenia.

Our primary objectives were to: (1) Identify the optimal model architecture to explain MEG data in the frequency domain, focusing on thalamo-cortical connectivity. (2) Quantify inter-population connectivity differences between schizophrenia patients and healthy controls. (3) Perform synaptic restoration analysis to determine synaptic and connectivity changes needed to shift schizophrenia models toward healthy controls.

The restoration analysis provides a pathway to understand how neural circuits in schizophrenia might be ”corrected” to resemble functional patterns of healthy individuals. This approach extends beyond identifying dysfunction to simulating synaptic adjustments across key neurotransmitter systems. By restoring balanced connectivity, this analysis provides insights into the complex interactions between receptor systems that could theoretically align neural patterns in schizophrenia, advancing our understanding of the mechanisms underlying cognitive and perceptual disturbances.

## 2 Methods and Materials

### 2.1 Study Design & Participants

The present study applied computational modeling to MEG data collected and analyzed by Shaw et al.,^15^ which examined visual gamma responses in schizophrenia patients and healthy controls. Their experimental methodology can be found in the original paper^15^ and in the Supplementary materials (Section 4.7), and is briefly summarized below to contextualize our modeling approach.

#### 2.1.1 Study Population

The dataset comprised 28 individuals with schizophrenia recruited through the Cognition in Psychosis study^27^^,^^28^ and 30 healthy controls recruited from Cardiff University. All procedures were approved by the South East Wales NHS Ethics Board and Cardiff University’s School of Psychology Ethics Board.

#### 2.1.2 Selection Criteria

Participants (aged 16-75 years) were English-speaking with normal/corrected vision. Exclusion criteria included current substance abuse (verified by MINI interview^29^), epilepsy, major neurological incidents, and metallic implants. Controls were additionally excluded if they or first-degree relatives had psychiatric diagnoses.

#### 2.1.3 Visual Gamma Paradigm

The visual gamma paradigm presented a centrally positioned circular sine wave grating (5 $\deg $ diameter, 2 c.p.d. spatial frequency) contracting at 2.2 $\deg $/second with random speed variations. Participants completed 3 80-trial runs, responding to speed changes via button presses.

#### 2.1.4 MEG Recording and Preprocessing

MEG recordings were conducted using a 275-channel Canadian Thin Films (CTF) system at 1200 Hz sampling rate. Data were segmented into 4-second trials centered on stimulus onset. Synthetic aperture magnetometry (SAM) beamformer analysis^30^ compared brain activity between 2-second baseline and post-stimulus periods within the 30-80 Hz gamma band. Time-series were reconstructed from each participant’s peak voxel identified in the SAM gamma-band image.

For each participant, we identified a representative cortical source based on visually induced gamma activity. We then estimated the synaptic parameters of a thalamocortical model that best explained the power spectral density of this source during visual stimulation.

### 2.2 Computational Modeling

#### 2.2.1 Overview of the Thalamo-Cortical Model

We employed dynamic causal modelling (DCM)^5^ for steady-state responses to analyze the spectral densities of the MEG signals. This approach utilizes parameterized dynamical systems models; specifically state-space formulations of differential equation models (*neural masses*), which describe the evolution of variables of interest (eg, membrane potentials) as a function of connectivity parameters.^5^ The thalamo-cortical (TCM) model incorporates interacting, layer-resolved cortical and thalamic populations, based on the architectures proposed by Douglas and Martin^31^ and earlier work by Gilbert and Wiesel^32^.

The TCM model^22^ is comprised of conductance-based equations of the forms of Hodgkin and Huxley and, subsequently, Morris and Lecar.^33^ The model includes pyramidal and interneuron populations in cortical layers 2/3 and 5, a stellate cell population in layer 4, a thalamic-projection pyramidal cell population in layer 6, and reticular and relay populations in the thalamus.^34^

The dynamics of each population are determined by coupled differential equations:


(1) \begin{align*} & C\frac{dV}{dt} = \sum g_{n}(V-V_{n}) + u \end{align*}

(2) \begin{align*} & \dot{g}_{n} = \kappa_{n}(\varsigma_{n} - g_{n}) \end{align*}

(3) \begin{align*} & \varsigma = \gamma_{i,j}\sigma(\mu_{j} v - V_{R}, \Sigma_{j}) \end{align*}

Here, $ V $ represents membrane potential, $ g_{n} $ denotes conductance change due to receptor $ n $, $ V_{n} $ is the reversal potential of channel $ n $, $ C $ is the membrane capacitance, and $ u $ includes external or internal input currents. $ \kappa _{n} $ is the decay rate for channel $ n $, $ \gamma _{i,j} $ is the coupling parameter between populations $ i $ and $ j $, and $ \sigma $ represents the sigmoid function converting membrane potential into population firing rates.

Note that the equations of this neural mass model are nonlinear in the latent neuronal states (ie, voltages and conductances). This nonlinear class of conductance-based models affords a biophysical realism and interpretability—in contrast with linear convolution models—that allows one to model things like voltage dependent depolarization.

**Figure 1:**
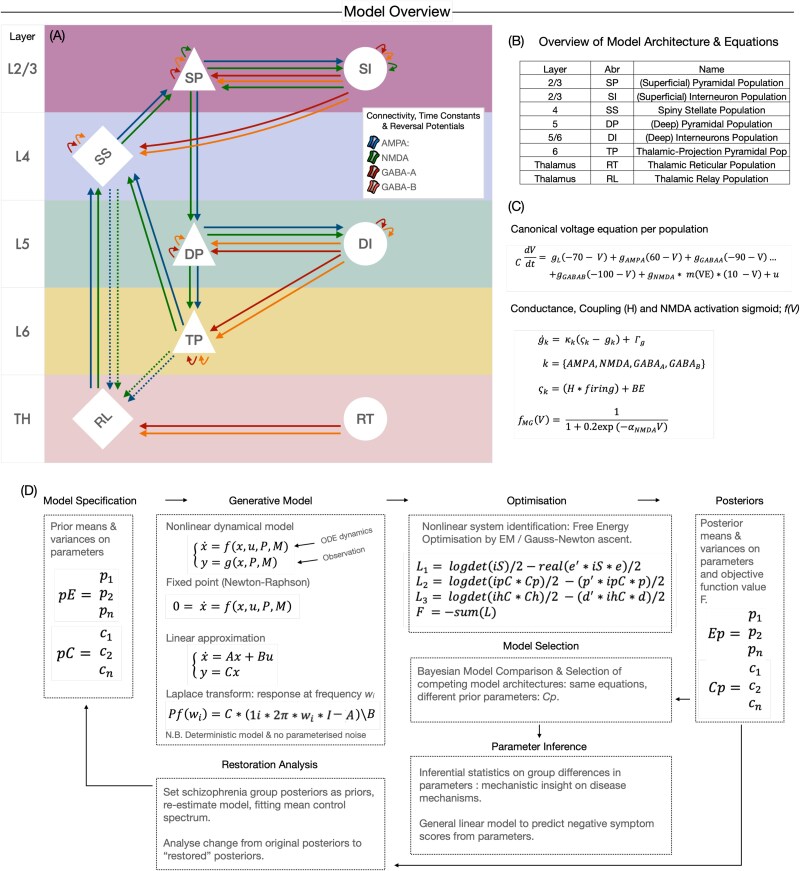
Thalamo-Cortical Model Architecture and Population Dynamics. (A) Schematic overview of the thalamo-cortical model architecture showing the connectivity between different neural populations across cortical layers (L2/3 to L6) and thalamic regions (TH). (B) Table summarizing the abbreviations for each population and their corresponding layers in the thalamo-cortical circuit. (C) The canonical voltage equation per population defines the dynamics of the membrane potential $V$, which includes contributions from leak conductance ($g_{L}$), AMPA (2.2 ms delay), GABA-A (5 ms delay), GABA-B (300 ms delay), and NMDA (100 ms delay) receptor-mediated currents. Thalamic relay and layer 6 pyramidal populations further posses non-inactivating potassium channels (Kv7 or M-channels, 160 ms) and hyperpolarization-activated cation currents (H-channels, 100 ms). The nonlinear activation profiles of NMDA and H channels are modeled with sigmoid and inverse-signomid functions, respectively. NMDA conductance is modulated by voltage-dependent magnesium block represented by $f_{MG}(V)$. (D) The process begins with model specification, where prior means ($pE$) and variances ($pC$) on the parameters are defined. The generative model uses a nonlinear dynamical system represented by ordinary differential equations (ODEs), and applies fixed point and linear approximations to solve the system, including Laplace transform for response at frequency $\omega _{i}$. In the optimization stage, the system undergoes parameter estimation using free energy (a variational bound on log marginal likelihood or model evidence) optimization via expectation maximization (EM) or Gauss-Newton ascent. The resulting posterior means ($Ep$) and variances ($Cp$) on the parameters are used for model selection, where Bayesian model comparison selects the best fitting model architecture. Parameter inference involves statistical analysis on group differences in parameter estimates, providing mechanistic insights into disease mechanisms. In a final step, restoration analysis, we re-estimate parameters using schizophrenia group posteriors and analyze differences with the control group.

Our model incorporates various conductance channels: AMPA, NMDA, GABA$_{A}$, GABA$_{B}$, M, and H (Figure 1A). M and H channels are exclusive to layer 6 thalamic-projection pyramidal cells and thalamic relay populations, which facilitate their characteristic bursting behavior. Our model is an extention of the NMDA model by Moran et al.,^35^ which includes a voltage-dependent mechanism for the NMDA magnesium block, with parameterized exponent a:


(4) \begin{align*}& f_{\textrm{MG}}(V) = \frac{1}{1 + 0.2\exp(-\alpha_{\textrm{NMDA}}V)}\end{align*}

Reversal potentials and decay rates were adopted from established models and literature to enhance physiological accuracy, including specific parameters for GABA$_{B}$, M-channels, and H-channels^36–38^ (Figure 1C). M-channels contribute to intrinsic cell membrane dynamics and excitation-inhibition balance, while H-channels influence resting membrane potential and may contribute to macroscopic network oscillations.^39–42^ GABA$_{B}$ receptors have been linked to macroscale oscillations, particularly in the gamma range.^43^^,^^44^

#### 2.2.2 Transfer Function

In accordance with other spectral DCM approaches, we employed the Laplace transform to compute the power spectrum of the model. The full procedure is outlined in (Figure 1D). Briefly, we performed a local linearization by numerically computing the Jacobian of the system about a fixed point. From this linearized approximation of the model, the Laplace transform at frequency i, in hertz, is given by:


(5) \begin{align*} & {H(s) = C \left( sI - A \right)^{-1} B} \end{align*}

(6) \begin{align*} & {\mathcal{L}\ (\omega_{i})\ = C(1\mathbf{i}\ast2\pi\ast \omega_{i}\ast I-A)^{-1} B} \end{align*}

Here (5) denotes the “canonical form” Laplace transform of a system, where *H(s)* is the transfer function and *s* is the complex Laplace variable. Equation (6) represents the Laplace transform evaluated at a specific frequency, *w(i)*, in hertz. A is the system Jacobian matrix (*df/dx*), B the input Jacobian (*df/du*), and C a vector of (fixed) weights controlling the contribution of each state element to the output local field potential. This transfer function approach is computationally efficient compared with numerically integrating the model equations. In contrast to conventional spectral DCM, we did not incorporate any spectral noise components explicitly, such as 1/f ”aperiodic” components or a discrete cosine basis set to represent neuronal fluctuations in the frequency domain. Thus, the model output comprises only the Laplace transform of the model fitted to data under the assumption of a white noise input.

Generally speaking, dynamic causal modelling for cross spectral density rests upon driving neural mass models with endogenous fluctuations from unmodelled sources that typically have a 1/f form. The fact that we were able to obtain good explanations for our empirical spectra suggests that the thalamocortical model used in this work was sufficiently expressive to reproduce a 1/f spectrum (see Results, Figure 3B) without parameterizing the contribution of unmodelled sources in the usual way.

**Figure 2:**
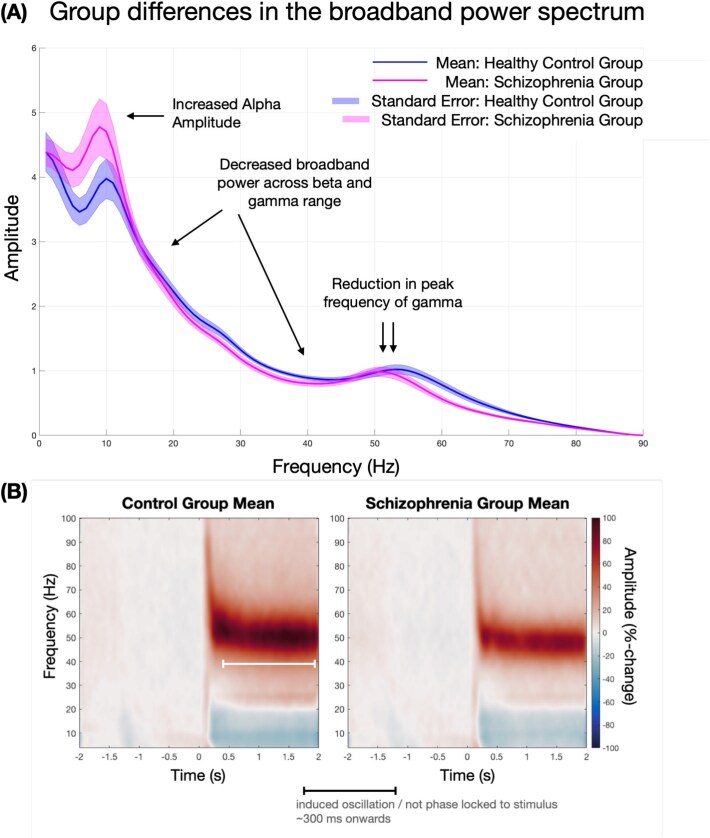
Comparison of Brain Activity Patterns Between Healthy Controls and Individuals with Schizophrenia. (A) Group differences in the broadband power spectrum. The graph shows mean amplitude across frequencies for healthy controls (blue) and schizophrenia patients (pink), with standard error bands. Key differences include increased alpha amplitude, decreased broadband power across beta and gamma ranges, and reduced peak frequency of gamma in the schizophrenia group. (B) Time-frequency representations of mean brain activity for control and schizophrenia groups. The heatmaps display frequency (*y*-axis) over time (*x*-axis), with color intensity representing amplitude changes. An induced oscillation period is noted, occurring approximately 300 ms onwards and not phase-locked to the stimulus. The schizophrenia group shows altered patterns of activity, particularly in higher frequency ranges, compared to controls. These spectral differences, first reported by Shaw et al.,^15^ form the basis for the modeling work presented in this paper.

The TCM implementation contains 56 variables consisting of 8 populations, with 7 states each: $ \textrm{mV}, g_{\textrm{AMPA}}, g_{\mathrm{GABA-A}}, g_{\textrm{NMDA}}, g_{\mathrm{GABA-B}}, g_{M}, $, and $ g_{H} $.

The cortex-thalamus-cortex feedback loop in the TCM incorporates a parameterized delay, initially set to 11 ms (comprising 8 ms from cortex to thalamus and 3 ms from thalamus to cortex). This delay was estimated during model fitting to accommodate discrepancies in literature-based estimates.^45–47^ Model priors can be found in Table 2 in Section 4.8 of the Supplementary materials.

### 2.3 Model Selection

We compared alternative thalamo-cortical models using Bayesian model comparison,^48^^,^^49^ which balances goodness-of-fit against complexity via the log-evidence (free-energy) approximation.^50^ The model space contained 15 nested variants of the thalamo-cortical microcircuit (TCM; Figure S6, Supplementary materials, Section 4.9): 4 single-receptor models (AMPA, NMDA, GABA-A, GABA-B), 6 pairwise combinations, 4 3-receptor combinations, and the full 4-receptor model.

Log-evidence was summarized with fixed-effects (FFX) and random-effects (RFX) Bayesian model selection. RFX outputs included the expected model frequency, exceedance probability, protected exceedance probability, and the Bayesian omnibus risk (BOR), computed for (1) all datasets, (2) schizophrenia datasets, and (3) control datasets.

To gauge the reliability of parameter estimates, we performed a parameter-recovery analysis on the winning model (Supplementary materials, Section 4.11). Several parameters showed poor recovery (intraclass correlation coefficient, ICC ¡ 0.4^51^). We therefore fixed these parameters at their prior means and re-estimated a reduced DCM. The reduced model was used as a robustness check in the subsequent parameter-based analyses (see Sections 2.4.1 and 2.4.3); unless otherwise stated, all reported results refer to the original (full) model. Numerical details, including *R*^2^; and BIC, are provided in the Supplementary materials (Section 4.9).

### 2.4 Parameter-Based Analyses

#### 2.4.1 Between-Group Parameter Differences

We applied the TCM to individual participant data, generating a set of parameter estimates for each participant. To assess group differences between healthy controls and individuals with established schizophrenia, we employed randomization-based post hoc testing with omnibus correction,^52^ using 5000 randomizations. This approach provides robust control of family-wise error rate when performing multiple comparisons across model parameters. This analysis was repeated for the reduced model (with fixed parameters for parameters showing ICC ¡ 0.4). Additionally, we performed a complementary pParametric mpirical Bayes analysis, detailed in the Supplementary materials (Section 4.12).

For parameters showing significant group differences, we conducted a contribution analysis to examine their individual effects on spectral outputs (Supplementary materials, Section 4.14). The relationship between model parameters and negative symptom severity was examined using stepwise regression analysis (Section 4.15 of the Supplementary materials).

#### 2.4.2 MEG-Parameter Group Classification

Building on our previous work distinguishing individuals with schizophrenia from healthy controls using MEG signals,^15^ we sought to determine whether model parameters from the thalamocortical model could provide additional mechanistic insight. To this end, we conducted a principal component analysis (PCA) on both the MEG power spectra and the estimated model parameters. The first principal component from the parameter set and the second from the power spectral density were used to assess group separation. We further examined the parameter loadings on the principal components to identify which specific parameters contributed most to group differences, thereby enabling exploration of potential mechanistic distinctions between schizophrenia and control groups. This multivariate approach was chosen to capture complex patterns in the data that may not be apparent in univariate analyses.

#### 2.4.3 Parameter Restoration Analysis

To quantify the synaptic changes required to shift the neurophysiological signature of schizophrenia toward that of healthy controls, we performed a parameter ”restoration” analysis. This approach quantifies the extent to which parameters from the schizophrenia group need to be adjusted to match the spectral characteristics observed in the control group. This analysis was done for the full and reduced model separately.

Initially, we set the posterior parameter estimates obtained from the schizophrenia group as prior distributions for a new model estimation. Utilizing these priors, we re-estimated the TCM by fitting it to the mean spectrum of the control group, resulting in a set of “restored” posterior parameters. Like the original model fits, this re-optimization also employed the variational Laplace routine in DCM, which optimizes a free energy objective function. Therefore, this restoration analysis assumes that the movement in synaptic connectivity necessary to move the neurophysiological signature of an individual with schizophrenia to that of the mean control individual, follows the path described by minimizing free energy.

We analyzed the changes from the original schizophrenia group posteriors to these restored posteriors to understand the parametric differences that account for the spectral disparities between the 2 groups. To quantify the significance of these changes, we employed randomization-based post hoc testing with omnibus correction,^52^ using 5000 randomizations. This analysis quantifies which TCM parameter adjustments would theoretically be required to shift the spectral characteristics of the schizophrenia group to match those of healthy controls.

## 3 Results

### 3.1 Spectral Power Responses

As previously reported by Shaw et al.,^15^ analysis of the broadband power spectrum revealed significant differences between the schizophrenia group and healthy controls (Figure 2A) [*F*(1, 29) = 4.15, *P* =.047] with the SZ group showing an estimated mean reduction of 3 Hz (controls mean = 58, SE = 0.92, SZ mean = 55, SE = 0.95). Most notably, the schizophrenia group exhibited an increased alpha amplitude compared to the control group, with a prominent peak observed in the 8-12 Hz frequency range. This elevation in alpha power was particularly pronounced around 10 Hz. In contrast to the alpha band, the schizophrenia group showed decreased broadband power across the beta (13-30 Hz) and gamma (30-80 Hz) ranges relative to healthy controls. This reduction in high-frequency power was accompanied by a subtle shift in the peak frequency of gamma oscillations, with the schizophrenia group displaying a lower peak frequency compared to controls.

Additionally, the time-frequency analysis highlighted reduced induced high-frequency oscillations in the schizophrenia group, particularly in the gamma range, consistent with the decreased broadband power observed in the spectrum analysis.

### 3.2 Computational Modeling Results

These spectral differences, originally identified by Shaw et al.,^15^ are modeled in this paper to determine which parameter differences best explain them through thalamo-cortical dynamics.

#### 3.2.1 Bayesian Model Comparison

We evaluated 15 variants of the thalamo-cortical model across the full cohort and within the schizophrenia and control groups separately. In all 3 datasets, FFX analysis assigned the highest posterior probability to the fully connected model. RFX analysis, which accommodates inter-subject variability, yielded concordant results: the expectation of the posterior, exceedance, and protected exceedance probabilities all favored the full model, while BOR values were near zero, indicating that this preference was unlikely to have arisen by chance.

**Figure 3:**
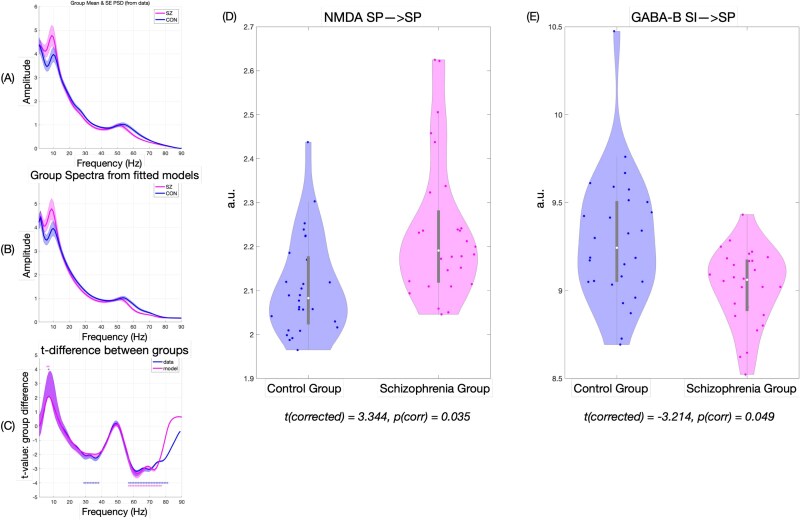
Spectral and Synaptic Parameter Comparison Between Control and Schizophrenia Groups. (A) Group spectra from MEG virtual sensor: This graph shows the group mean and standard error of the power spectral density (PSD) derived directly from MEG data for both SZ (pink) and CON (blue) groups across frequencies from 0 to 90 Hz. (B) Group spectra from fitted models: This graph displays the group mean and standard error of the PSD as predicted by the fitted computational models for both SZ and CON groups. The close resemblance to panel A indicates a good model fit for both groups. (C) To evaluate how well the model captured group differences, we computed t-statistics across frequencies comparing the spectral differences between groups in both the empirical data and model predictions. While these frequency-domain t-statistics exhibit serial correlations (adjacent frequency points are not independent), we maintained analysis across the full spectrum rather than reducing to discrete points like peaks and troughs. This approach allowed us to assess how well the model reproduced group differences across all frequencies, validating its ability to capture the complete spectral characteristics of both groups. The resulting t-statistics demonstrate the model’s success in reproducing the empirical group differences across the frequency range. Panels D and E are violin plots illustrating group differences in specific synaptic parameters: NMDA SP$\rightarrow $SP connectivity (D) shows higher values for the schizophrenia group, while GABA-B SI$\rightarrow $SP connectivity (E) shows lower values.

Together, these findings indicate that a densely connected thalamo-cortical circuit provided the best explanation of the MEG data in both healthy controls and individuals with schizophrenia. Comprehensive BMC outputs—including variance explained (Figure S7), FFX and RFX summaries (Figure S8), and BIC distributions for all 15 candidates (Figure S9)—are provided in the Supplementary materials (Section 4.9). (Figure 3B) shows the power spectra predicted by the winning model for each group, and (Figure 3C) presents the corresponding $t$-statistics. Results of the parameter-recovery analysis are reported in the Supplementary materials, Section 4.11.

### 3.3 Parameter-Based Analyses

#### 3.3.1 Parameter Comparison

Parameter comparison between the control and schizophrenia groups revealed 2 significant differences in the full model: The NMDA-mediated self-connection of superficial pyramidal (SP) cells was increased in schizophrenia ($t_{\textrm{corr}} = 3.344,\; p_{\textrm{corr}} = 0.035$), whereas the GABA$_{\textrm{B}}$ projection from superficial interneurons (SI) to SP cells was weaker ($t_{\textrm{corr}} = -3.214,\; p_{\textrm{corr}} = 0.049$). The complementary PEB analysis supported these findings and revealed additional group differences (see Supplementary materials, Section 4.12).

Repeating the analysis with a reduced model, that is, fixing parameters with $\mathrm{ICC} < 0.4$, again showed an NMDA SP-SP increase ($t_{\textrm{corr}} = 1.458,\; p_{\textrm{corr}} = 0.032$), but the GABA$_{\textrm{B}}$ SI-SP effect disappeared. Instead, GABA$_{\textrm{A}}$ SI-SP ($t_{\textrm{corr}} = -3.264,\; p_{\textrm{corr}} = 0.025$) and AMPA-mediated SP-SI ($t_{\textrm{corr}} = -2.303,\; p_{\textrm{corr}} = 0.047$) connections were found to be reduced in the schizophrenia grouped.

Contribution analyses of the 2 significant parameters from the full model (NMDA SP-SP and GABA$_{\textrm{B}}$ SI-SP) showed that manipulating each in isolation produced spectral changes that were smaller in magnitude and opposite in direction to the observed group differences (Figure S13).

#### 3.3.2 Group Separation Between Controls and Schizophrenia

We applied PCA to both the power spectra and full model parameters. Plotting the first principal component of the model parameters against that of the power spectra revealed a clear group separation in 2-dimensional space. Interestingly, the distribution of model parameters within each group was bimodal, a feature discussed further in Section 4.3. Examination of the principal component loadings (see Figure S15) showed that GABA-A self-connections on superficial inhibitory neurons (SI-SI) contributed most strongly and positively, while GABA-B parameters consistently loaded negatively, indicating an opposing influence of these receptor systems on the variance.

#### 3.3.3 Parameter Restoration

In the restoration analysis we quantified the synaptic adjustments needed for the schizophrenia model to reproduce the control power spectrum (Figure 5). In the *full* thalamo-cortical model, 20 connections needed significant adjustment (Figure 5A-B), spanning NMDA-, AMPA-, GABA$_{\textrm{A}}$-, and GABA$_{\textrm{B}}$-mediated pathways.

**Figure 4:**
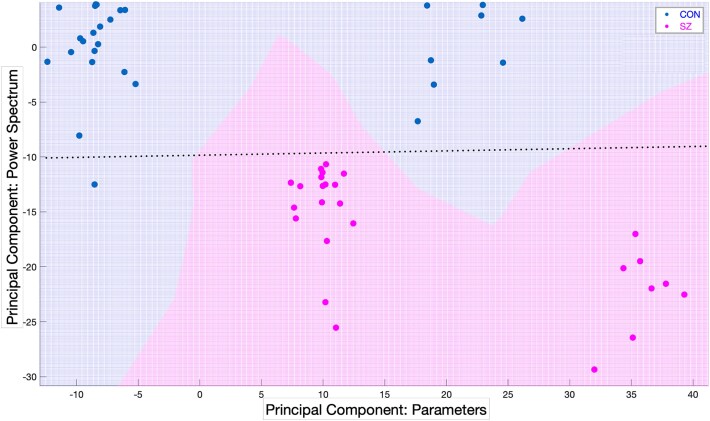
Principal Component Analysis of Power Spectra and Model Parameters. The *x*-axis represents the principal component derived from model parameters, while the *y*-axis shows the principal component from power spectrum data. Blue dots (mainly located on the left and upper side) indicate control subjects, and pink dots (bottom.right) represent individuals with schizophrenia. The plot demonstrates clear separation between the 2 groups.

**Figure 5:**
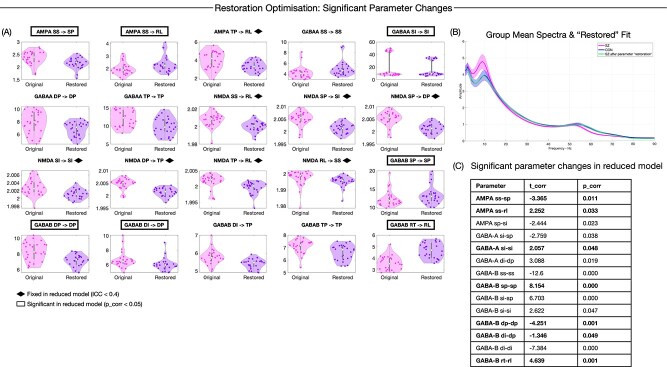
Restoration Optimization: Significant Parameter Changes and Spectral Effects. (A) Violin plots show changes in synaptic parameters between the original schizophrenia group (pink) and the computationally ”restored” state (purple). Each plot represents a different synaptic connection. Most importantly, more parameters needed to be adjusted significantly to match the power spectrum of the healthy controls than differed significantly between schizophrenia and healthy controls in our initial analysis. (B) Power spectra comparison showing the original control group (blue), schizophrenia group (pink), and the ”restored” schizophrenia model (green). The *x*-axis represents frequency (Hz), and the *y*-axis represents amplitude. (C) Table representing the significantly different parameters performing the restoration on the reduced model (fixed parameters with ICC ¡ 0.4). Parameters in bold were found to be significantly different in both the full and reduced model.

Repeating the procedure with the *reduced* model (where parameters with low recoverability ($\mathrm{ICC}<0.4$) were fixed) identified 14 significant connections (Figure 5C). Seven of these overlapped with the full-model results.

## 4 Discussion

In this study, we employed a thalamo-cortical conductance-based model to investigate neural circuit dynamics in schizophrenia using MEG data. Our approach combined spectral analysis, dynamic causal modelling, and parameter restoration.

### 4.1 Spectral Analysis

The findings, originally reported by Shaw et al.^15^ revealed distinct alterations in neural oscillatory patterns in schizophrenia. The most prominent observation was the significantly increased alpha (8-12 Hz) power in the schizophrenia group relative to healthy controls. This may suggest an impairment in alpha modulation, potentially related to deficits in attentional control and sensory gating that are frequently observed in schizophrenia.^53^ In contrast to the heightened alpha power, there was decreased power in the beta (13-30 Hz) and gamma (30-80 Hz) frequency ranges in the schizophrenia group. This finding is consistent with previous research,^14^^,^^15^^,^^16^^,^^17^ which provided robust evidence for reduced gamma-band activity across various paradigms in schizophrenia. Our results extended this finding to the beta range, suggesting broader deficits in high-frequency oscillations. A more subtle but noteworthy observation is the reduction in peak gamma frequency in the schizophrenia group, consistent with findings in the literature.^54^^,^^55^^,^^56^

Crucially, it is only by employing computational modelling across these broadband spectral group differences, that we are able to investigate the underlying circuit mechanisms that contribute to these observed neural differences in the schizophrenia pathophysiology.

### 4.2 Parameter Inference and Excitation/Inhibition Imbalance

Our analysis identified 2 key group differences in the full model: increased NMDA-mediated recurrent excitation among superficial pyramidal (SP) cells ($t_{\textrm{corr}} = 3.344, p_{\textrm{corr}} = 0.035$) and decreased GABA-B-mediated inhibition from superficial interneurons (SI) to SP cells ($t_{\textrm{corr}} = -3.214, p_{\textrm{corr}} = 0.049$). These effects appeared in both the primary and PEB analyses (see Supplementary materials, Section 4.12), and extend our previous findings with a simpler model.^15^

Given concerns about parameter identifiability (see Section 4.6), we repeated the analysis using a reduced model in which all parameters with $\mathrm{ICC}<0.4$ were fixed. In this reduced model, the increase in NMDA SP-SP excitation remained significant ($t_{\textrm{corr}}=1.458, p_{\textrm{corr}}=0.032$), while the GABA-B SI-SP effect was no longer observed. Instead, we identified significant reductions in GABA-A SI-SP ($t_{\textrm{corr}}=-3.264, p_{\textrm{corr}}=0.025$) and AMPA SP-SI ($t_{\textrm{corr}}=-2.303, p_{\textrm{corr}}=0.047$) connections.

The reduction in AMPA SP-SI connectivity suggests decreased excitatory input to inhibitory interneurons, which may result in less activation of these inhibitory cells and, consequently, reduced inhibition; a form of circuit disinhibition. Together with the observed changes in NMDA and GABAergic signaling, these findings point toward a superficial-layer E/I imbalance in schizophrenia.

Notably, the observed increase in NMDA-mediated excitation contrasts with the classic NMDA hypofunction hypothesis,^57–60^ which posits reduced NMDA function in chronic schizophrenia.

Several interpretations of this result are possible:

1. **Hyperfunction/Hypofunction:** Our results bear a close resemblance to the hyperglutamatergic state often observed in first-episode schizophrenia or individuals at clinical high-risk of psychosis. Early stages of the disorder are characterized by increased glutamate (potentially representing NMDA receptor hyperfunction), which later transitions to reduced glutamate (NMDA hypofunction), as a compensatory mechanism, in chronic stages.^61–64^ While our sample consists of individuals with established schizophrenia, this finding raises the possibility that some individuals might retain or revert to a NMDA hyperfunction. This may suggest a more dynamic and heterogeneous course of glutamatergic dysfunction in schizophrenia than previously thought.
2. **Compensatory Mechanism:** The observed increase in NMDA self-connection could potentially represent a localized compensatory response to NMDA receptor hypofunction elsewhere in the brain. However, this interpretation is problematic. Previous studies have consistently shown decreased glutamate in primary sensory areas, including V1, in schizophrenia.^59^
3. **Ketamine and Gamma Activity:** The findings from Uhlhaas et al.^65^ suggest that ketamine, an NMDA receptor antagonist, increased visual gamma activity, which contrasts with the decreased gamma activity typically observed in schizophrenia. This suggests that NMDA receptor dysfunction in schizophrenia may not simply involve hypofunction but could reflect a more complex state, potentially explaining the increased NMDA-mediated excitation observed in our study.

The reduction in GABAergic inhibition aligns with widespread evidence for inhibitory deficits in schizophre-nia.^66–68^ Together, these changes suggest a shift toward cortical hyper-excitability, particularly in superficial layers where NMDA, AMPA, and GABAergic mechanisms.

While these parameter differences were statistically significant, their interpretation should be tempered by the complexity of the model and potential parameter interactions (see Section 4.13, Supplementary materials), as well as the limitations in parameter recovery.

### 4.3 Principal Component Analysis and Parameter Distributions

PCA of model parameters revealed a bimodal distribution in both control and schizophrenia groups. Although PCA did not improve group separation beyond what was observed in the power spectral density, it provided complementary insights into the parameter landscape, particularly highlighting an opposing effect of GABA$_{A}$ and GABA$_{B}$ on the variance explained.

GABA$_{A}$ self-connections within superficial inhibitory neurons (si-si) showed the strongest positive loadings, while GABA$_{B}$ parameters loaded negatively, suggesting a trade-off between these receptor systems in inhibitory circuit function (Supplementary materials, Section 4.16). This pattern aligns with the principle of degeneracy, where different mechanistic configurations can yield similar functional outcomes.^69^ The consistent bimodal distribution across both groups indicates this is a fundamental organizational property of cortical inhibitory circuits, rather than a disease-specific effect. These 2 parameter configurations likely reflect natural variation in how inhibitory balance is achieved in the cortex, possibly representing alternative stable solutions shaped by development or genetics. Such configurations may produce similar spectral characteristics through different parameter combinations, underscoring the complex, non-linear mapping between model parameters and neural activity. Future research could explore whether these parameter modes relate to other neurophysiological or cognitive measures, further clarifying their functional significance.

### 4.4 Parameter Restoration

The restoration analysis indicated that shifting the schizophrenia model toward control-like spectral features required coordinated changes across multiple synaptic pathways and receptor systems. While the specific set of parameters requiring adjustment differed between the full and reduced models (with partial overlap), the overall pattern was consistent: restoration was not achieved by altering a single connection or receptor type, but instead involved addressing multiple synaptic mechanisms in concert.

However, for practical therapeutic development, a more focused approach would consider coordinated changes across all parameters associated with specific receptor types, as pharmacological interventions typically modulate entire receptor systems simultaneously rather than individual, or laminar specific, synapses.

### 4.5 Therapeutic Implications and Future Directions

The complexity revealed by our restoration analysis has important implications for therapeutic development. While previous studies have identified E/I imbalance in schizophrenia, our computational approach provides preliminary evidence that circuit dysfunction cannot be reduced to simple increases or decreases in single neurotransmitter systems. Instead, our results suggest that effective therapeutic strategies might need to move beyond traditional single-target approaches to address the coordinated alterations across multiple neurotransmitter systems we observed.

To translate these insights into therapeutic applications, future work should leverage computational simulations to systematically explore how different combinations of interventions might restore healthy circuit function. This approach could help identify promising multi-target treatment strategies before proceeding to clinical trials.

### 4.6 Limitations and Interpretational Considerations

First, parameter recovery analysis indicated that a substantial proportion of model parameters, particularly NMDA receptors, were not reliably identifiable from the available data. To address this, we repeated key analyses using a reduced model in which poorly recoverable parameters were fixed. While the main conclusions regarding excitation-inhibition imbalance and the need for distributed synaptic changes were consistent across models, the specific parameters implicated varied depending on model constraints.

Second, the non-linear mapping between model parameters and spectral output means that different combinations of parameter changes can produce similar spectral features. While realistic of biological systems, this degeneracy complicates direct mechanistic interpretation and suggests caution in attributing group differences or restoration effects to individual synaptic pathways.

Finally, our results are based on a specific thalamo-cortical model and MEG dataset; generalizability to other models, brain regions, or modalities remains to be established. Future work should aim to improve parameter identifiability, incorporate multimodal data, and validate findings using pharmacological designs.

## Supplementary Material

supplementary_rev3_sbaf149

## Data Availability

This study did not generate any new data. All analyses were conducted on previously published data by Shaw et al.^15^
