# Supplementary material for: Restoring Synaptic Balance in Schizophrenia: Insights From a Thalamo-Cortical Conductance-Based Model
Source: Schizophr Bull. 2025 Sep 11;52(4):sbaf149. doi: 10.1093/schbul/sbaf149 (PMC13391648; doi:10.1093/schbul/sbaf149)
Supplement: supplementary_rev3_sbaf149 [file supplementary_rev3_sbaf149.pdf]

Supplementary Materials

4.7 Original Data Collection & Analysis

Here we are summarizing the procedure of data collection and analyses as performed by Shaw et al. (2020)[15] in more detail.

4.7.1 Participant Sample and Recruitment

Participants included 28 individuals diagnosed with schizophrenia (8 female, mean age: males = 44.2 years [SD = 8.6], females = 45.9 years [SD = 8]) and 30 healthy control participants (11 female, mean age: males = 42.4 years [SD = 10], females = 40.6 years [SD = 10.2]). The age range for the SZ group was 22-58 years, while the control group ranged from 24-58 years. There were no significant age differences between the groups ($P > .1$). Participants were recruited from the Cognition in Psychosis study (Schizophrenia Working Group of the Psychiatric Genomics Consortium) with recruitment details provided in Lynham et al. (2018)[28]. Controls were recruited primarily through an advert placed on the Cardiff University Noticeboard system and opportunistically from CUBRIC, Cardiff University. Ethical approval was obtained from the NHS Ethics Board and Cardiff University School of Psychology Ethics Board.

4.7.2 Inclusion and Exclusion Criteria

Inclusion criteria for SZ participants required a DSM-IV schizophrenia diagnosis, age 16-75 years, English as a first language, and normal or corrected vision. Healthy controls were required to meet the same age and language criteria and have normal or corrected vision. All participants provided informed consent and were screened for substance use and capacity to consent. Exclusion criteria included epilepsy, severe neurological events, or metallic implants. Controls were further excluded if they or a first-degree relative had a lifetime diagnosis of affective or psychotic disorders.

4.7.3 Participant Assessments

SZ participants were recruited through the Cognition in Psychosis study and assessed with the Schedule for Clinical Assessment in Neuropsychiatry (SCAN)[70] and clinical notes, with diagnoses confirmed using the MINI International Neuropsychiatric Interview[29]. SAPS[71] and SANS[72] ratings were used to assess symptom severity, and antipsychotic dosages were standardized using Olanzapine equivalents. Control participants underwent the MINI interview to confirm the absence of psychiatric disorders in themselves and first-degree relatives.

Group	Number	Mean Age (y)	SD Age (y)	Independent T-Test SZ Vs Controls (P value)
All controls	29	41.1	10.1	0.16
All SZ	27	44.7	8.5	
Female controls	11	40.2	12.1	0.3
Female SZ	8	45.9	8.0	
Male controls	18	41.6	9.4	0.4
Male SZ	19	44.2	8.8	

Table 1: Demographic characteristics of study participants. This table presents the number of participants, mean age, and standard deviation of age for control and schizophrenia (SZ) groups, further subdivided by gender. The rightmost column shows p-values from independent t-tests comparing age between SZ and control groups overall and within each gender. No significant age differences were found between SZ and control groups.

4.7.4 Data Collection and Participant Retention

One male control participant was unable to complete the MEG experiment. Additionally, two SZ participants (1 male, 1 female) did not complete MEG data collection. The final cohort included 29 controls and 27 SZ participants.

4.7.5 MEG Paradigm and Analysis

The MEG paradigm, based on Hoogenboom's approach[73], utilized centrally presented, circular sine wave gratings (diameter 5°, spatial frequency 2 cycles per degree, maximum contrast). The grating drifted inward toward a fixation point, with the speed increasing at a randomized interval (50-3000 ms after onset). Participants pressed a button upon detecting the speed change. Feedback on response accuracy ("OK,""early,""late") was provided during a 1000 ms resting period following stimulus offset.

MEG data were recorded using a 275-channel CTF MEG system at a sampling rate of 1200 Hz. Data were epoched into 4-second trials centered on stimulus onset and inspected for artifacts. Synthetic aperture magnetometry (SAM) beamforming analysis[30] was used to compare baseline (−2 to 0 s) and stimulation (0 to 2 s) activity. Volumetric SAM images in the 30-80 Hz range identified gamma activity peaks in the visual cortex. Virtual sensors were constructed at these peaks, and Hilbert[74] envelopes of bandpass-filtered data (1-100 Hz, 0.5 Hz steps) were used for time-frequency analysis. Responses were expressed as mean percentage changes from baseline. Peak frequency and amplitude features were derived from transient (0-300 ms) and sustained (300-800 ms) response windows.

Gamma power ($\geq$ 30 Hz) was analyzed across sessions to confirm that stimulus-induced gamma changes were not confounded by baseline gamma power differences. Minor uncorrected differences above this range were observed in session 1 ($t = 2.6$, $P = .014$) and session 3 ($t = 2.2$, $P = .034$).

The MEG visual grating paradigm included pseudo-random speed changes, which were excluded from analyses to avoid speed-related effects. Each experimental run comprised 80 trials, with participants completing three runs. Stimuli were presented using Presentation software and projected onto a screen within a magnetically shielded room, 60 cm from participants' eyes, at a refresh rate of 60 Hz.

4.8 Model priors

Name	Prior mean	Prior variance
AMPA_ss_to_sp	2	1/8
AMPA_ss_to_rl	2	1/8
AMPA_sp_to_si	2	1/8
AMPA_sp_to_dp	2	1/8
AMPA_dp_to_di	2	1/8
AMPA_dp_to_tp	2	1/8
AMPA_tp_to_ss	2	1/8
AMPA_tp_to_rl	2	1/8
AMPA_rl_to_ss	2	1/8

AMPA_rl_to_rt	2	1/8
GABAA_ss_to_ss	8	1/8
GABAA_sp_to_sp	18	1/8
GABAA_si_to_ss	10	1/8
GABAA_si_to_sp	10	1/8
GABAA_si_to_si	10	1/8
GABAA_dp_to_dp	8	1/8
GABAA_di_to_dp	6	1/8
GABAA_di_to_di	14	1/8
GABAA_di_to_tp	6	1/8
GABAA_tp_to_tp	8	1/8
GABAA_rt_to_rt	8	1/8
GABAA_rt_to_rl	8	1/8
GABAA_rl_to_rl	8	1/8
NMDA_ss_to_sp	2	1/8
NMDA_ss_to_rl	2	1/8
NMDA_sp_to_sp	2	1/8
NMDA_sp_to_si	2	1/8
NMDA_sp_to_dp	2	1/8
NMDA_si_to_sp	2	1/8
NMDA_si_to_si	2	1/8
NMDA_dp_to_di	2	1/8
NMDA_dp_to_tp	2	1/8
NMDA_tp_to_ss	2	1/8
NMDA_tp_to_rl	2	1/8
NMDA_rl_to_ss	2	1/8
NMDA_rl_to_rt	2	1/8
GABAB_ss_to_ss	8	1/8
GABAB_sp_to_sp	18	1/8
GABAB_si_to_ss	10	1/8
GABAB_si_to_sp	10	1/8
GABAB_si_to_si	10	1/8
GABAB_dp_to_dp	8	1/8
GABAB_di_to_dp	6	1/8
GABAB_di_to_di	14	1/8
GABAB_di_to_tp	6	1/8

GABAB_tp_to_tp	8	1/8
GABAB_rt_to_rt	8	1/8
GABAB_rt_to_rl	8	1/8
GABAB_rl_to_rl	8	1/8
Thalamo-Cortical delay	3 ms	1/8
Cortico-Thalamic Delay	8 ms	1/8
scale_NMDA	1	1/8
Electrode Gain	1	1/8

Table 2: Prior means and variances for thalamo-cortical and cortical-cortical synaptic parameters across different receptor types (AMPA, GABAA, NMDA, and GABAB) used during model fitting. Delays and gain parameters for thalamo-cortical and cortico-thalamic connectivity are also listed.

4.9 Bayesian Model Comparison

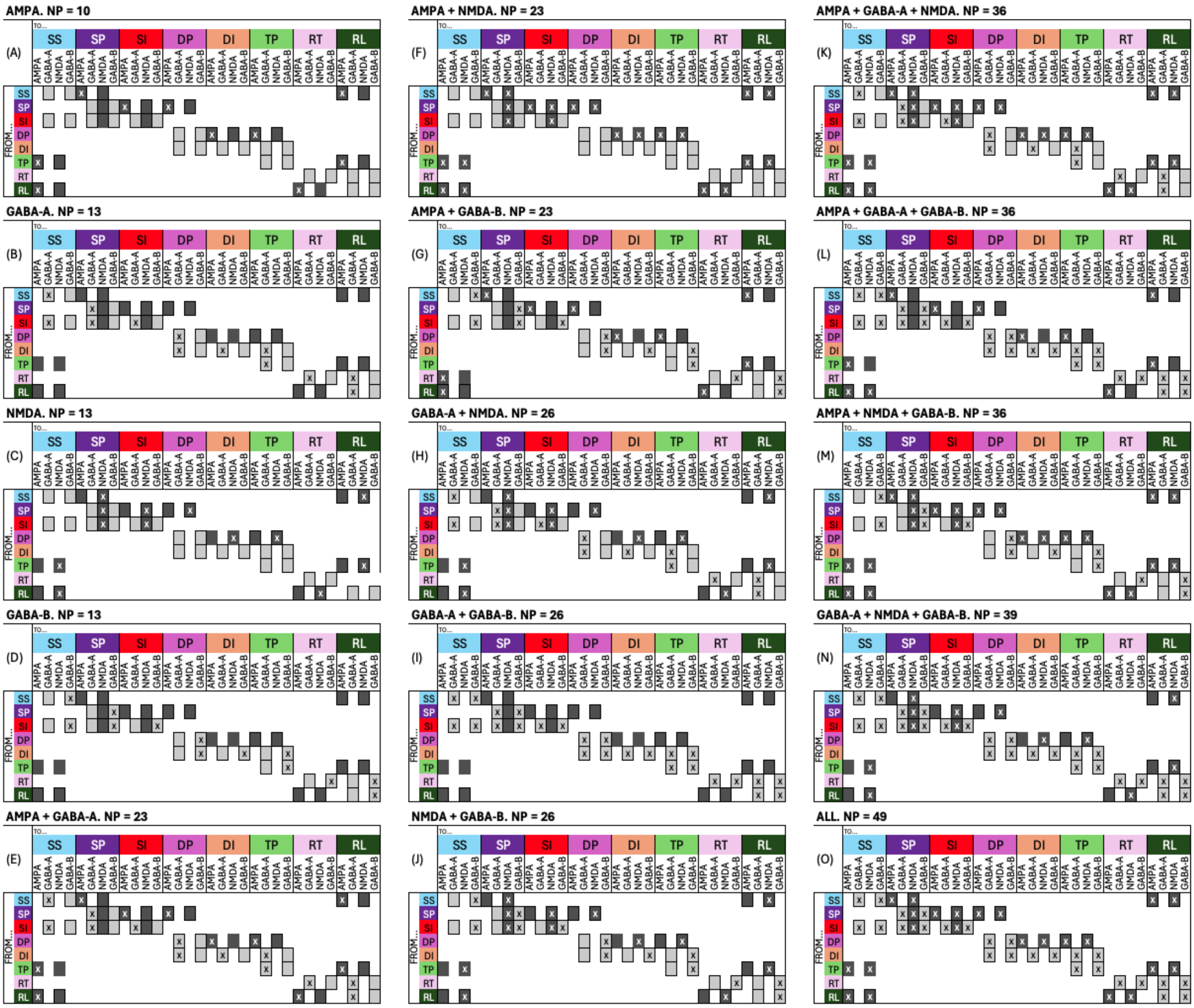

Figure 6: ***Connectivity matrices across different synaptic combinations.*** Each panel (A-O) represents the connectivity structure for a specific synaptic receptor combination: AMPA (**A**), GABA-A (**B**), NMDA (**C**), GABA-B (**D**), AMPA + GABA-A (**E**), AMPA + NMDA (**F**), AMPA + GABA-B (**G**), GABA-A + NMDA (**H**), GABA-A + GABA-B (**I**), NMDA + GABA-B (**J**), AMPA + GABA-A + NMDA (**K**), AMPA + GABA-A + GABA-B (**L**), AMPA + NMDA + GABA-B (**M**), GABA-A + NMDA + GABA-B (**N**), and all synaptic types (**O**). Each matrix illustrates the connections between neuronal populations (SP, SI, SS, DP, DI, TP, RL, RT) for each synaptic mechanism. Crosses within the squares denote existing connections that were allowed to vary during optimization whereas the absence of a cross denotes fixed connections during optimization. These submodels were subsequently used in the Bayesian Model Comparison. The dark grey boxes represent excitatory connections and light grey boxes represent inhibitory connections.

To systematically evaluate which combination of synaptic mechanisms best explains our MEG data, we performed Bayesian Model Comparison (BMC) across 15 different model variants. Each variant allowed different combinations of synaptic connections (AMPA, $GABA_A$, NMDA, and $GABA_B$) to vary during optimization while keeping others fixed (figure 6). This approach helped identify the most parsimonious model that balances complexity with accuracy in explaining the observed data.

The comparison was based on the Free Energy approximation to model evidence, which provides a principled way to compare models by considering both their accuracy in fitting the data and their complexity[50].

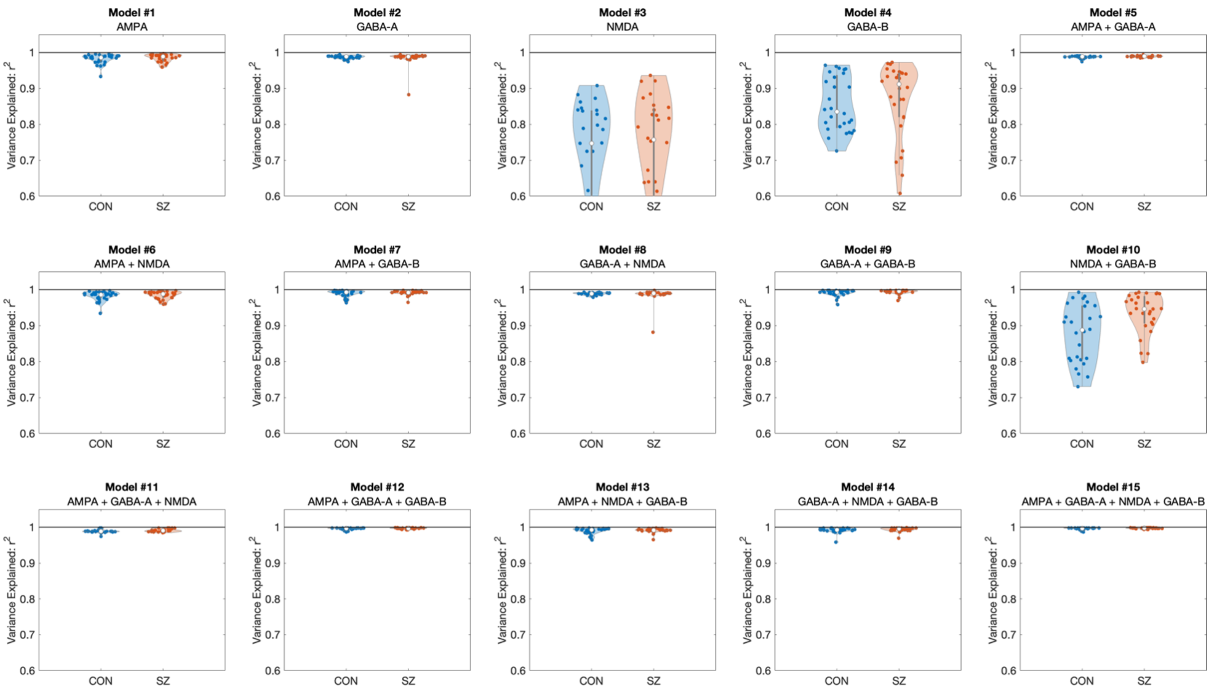

Figure 7: ***Model variance explained (R^2).*** Comparison of variance explained (R^2) between control (CON) and schizophrenia (SZ) groups across multiple models. Each subplot represents a different model, with the variance explained shown for both groups. Individual data points are overlaid, and violin plots in selected subplots illustrate the distribution of R^2 values within each group. The horizontal line at $R^2 = 1$ indicates the theoretical upper bound of variance explained.

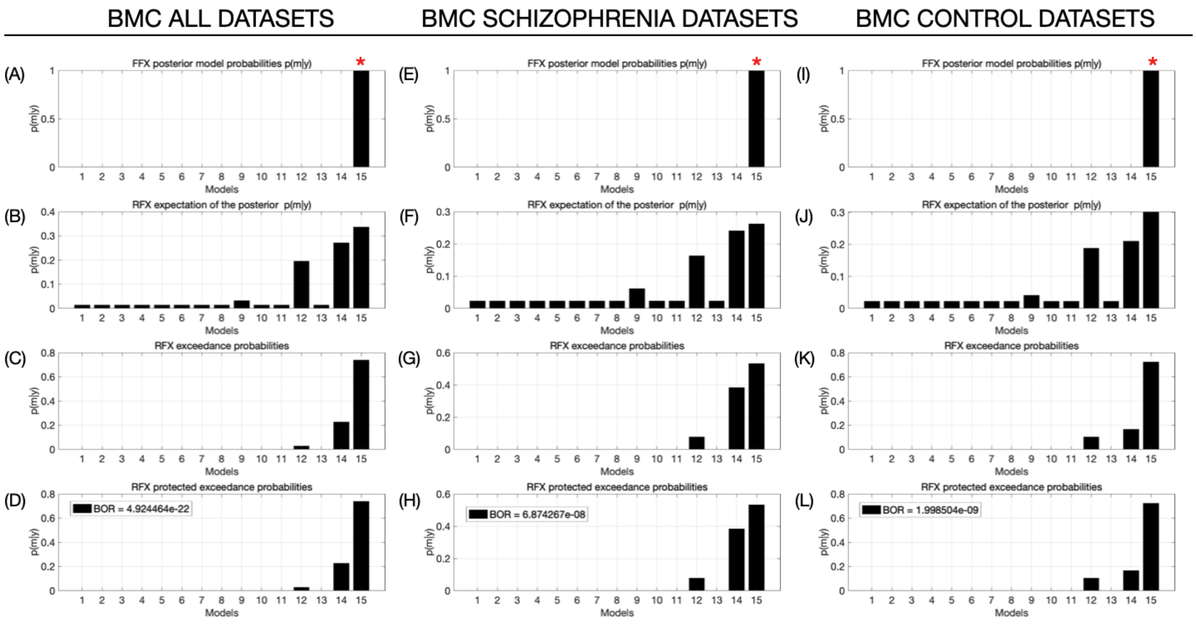

Figure 8: ***Fixed Effects (FFX) and Random Effects (RFX) Bayesian Model Comparison (BMC) analysis.*** The figure is organized into three main columns representing different dataset groupings: all datasets combined, schizophrenia datasets, and control datasets. Each column contains four rows of graphs (A-D, E-H, I-L) showing different aspects of the BMC analysis: **(A,E,I)** FFX posterior model probabilities: These graphs show the fixed effects analysis results, with model 15 consistently emerging as the winning model (marked with a red asterisk) across all dataset groupings. **(B,F,J)** RFX expectation of the posterior: These display the random effects analysis, showing the expected posterior probability for each model. Models 14-15 generally show higher probabilities, particularly in the control datasets. **(C,G,K)** RFX exceedance probabilities: These graphs indicate the probability that each model exceeds all others in the random effects analysis. Model 15 consistently shows the highest exceedance probability across all dataset groupings. **(D,H,L)** RFX protected exceedance probabilities: Similar to the exceedance probabilities, but protected against the possibility of all models being equally likely. The Bayes Omnibus Risk (BOR) values are provided, showing extremely low probabilities that all models are equally likely. This figure illustrates the results of both fixed and random effects analyses, consistently favoring model 15 across different analytical approaches and dataset groupings.

We conducted both FFX and RFX analyses to account for potential variability across subjects and to ensure robust model selection.

The model fit quality was additionally assessed using variance explained (R^2) metrics (figure 7) and Bayesian Information Criterion (BIC) distributions (figure 9), providing complementary evidence to our primary Free Energy-based comparison.

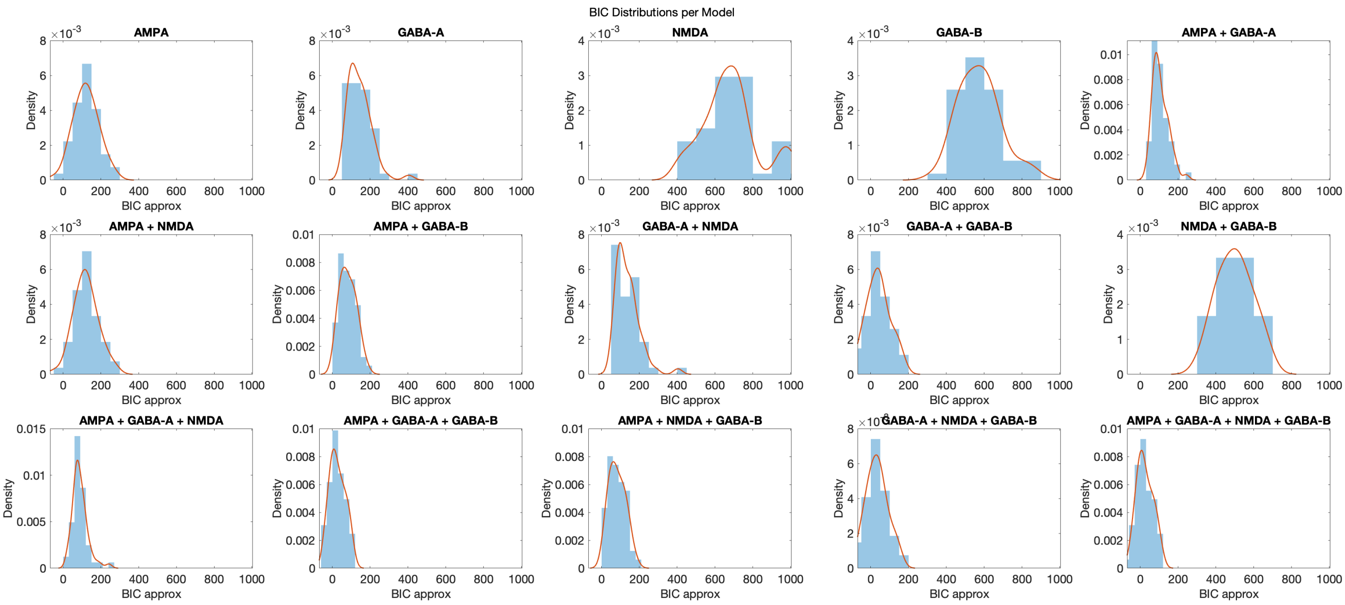

Figure 9: ***BIC distributions across model space*** BIC values for each of the 15 candidate models in our model space. Each subplot shows the histogram of BIC values (blue bars) with fitted probability density functions (red lines) across subjects. The x-axis represents BIC values (lower values indicate better model fit accounting for complexity), and the y-axis shows the density. These distributions help assess model performance while penalizing for model complexity.

4.10 Parameter Recovery

To assess the reliability of parameter estimation in our model, we conducted a parameter recovery analysis using Intraclass Correlation Coefficients (ICC). Initially, we found that many parameters in the full model were not reliably recoverable: of 50 parameters, 12 had no variance (remained at prior values), 14 showed poor recovery (ICC < 0.4[51]), 10 showed fair recovery (ICC 0.4–0.59), and only 16 showed good to excellent recovery (ICC > 0.6). Notably, the two parameters showing significant group differences; NMDA SP-SP and GABAB SI-SP, exhibited poor to fair recovery, limiting confidence in these specific findings.

To address this, we refitted the model with all parameters showing ICC < 0.4 fixed at their prior values, except for NMDA SP-SP, which was retained as a free parameter due to its importance in our primary results. We then repeated the parameter recovery analysis by re-fitting the model to predicted PSDs. In here, every free parameter achieved ICC > 0.5 and, apart from three parameters (AMPA SS-SP, GABAA SP-SP, GABAB SI-SI), all exceeded 0.6, indicating good-to-excellent reliability.

Parameter	ICC (Full model)	ICC (Reduced model)
AMPA_dp_to_di	0.000	Fixed
AMPA_dp_to_tp	**0.905**	**0.763**
AMPA_rl_to_rt	0.000	Fixed
AMPA_rl_to_ss	**0.753**	**0.696**
AMPA_sp_to_dp	**0.614**	0.675
AMPA_sp_to_si	**0.656**	**0.608**
AMPA_ss_to_rl	**0.892**	**0.842**
AMPA_ss_to_sp	**0.515**	**0.545**
AMPA_tp_to_rl	0.067	Fixed
AMPA_tp_to_ss	0.000	Fixed
CT	**0.705**	**0.776**
GABAA_di_to_di	**0.689**	**0.824**
GABAA_di_to_dp	**0.760**	**0.891**
GABAA_di_to_tp	**0.488**	**0.879**
GABAA_dp_to_dp	**0.752**	**0.739**
GABAA_rl_to_rl	0.000	Fixed
GABAA_rt_to_rl	**0.488**	**0.889**
GABAA_rt_to_rt	0.000	Fixed
GABAA_si_to_si	**0.749**	**0.626**
GABAA_si_to_sp	**0.538**	**0.695**
GABAA_si_to_ss	0.000	Fixed
GABAA_sp_to_sp	**0.521**	**0.578**
GABAA_ss_to_ss	**0.646**	**0.896**
GABAA_tp_to_tp	**0.728**	**0.763**
GABAB_di_to_di	0.177	Fixed
GABAB_di_to_dp	0.146	Fixed
GABAB_di_to_tp	0.053	Fixed
GABAB_dp_to_dp	0.228	Fixed
GABAB_rl_to_rl	0.000	Fixed
GABAB_rt_to_rl	**0.690**	**0.695**
GABAB_rt_to_rt	0.000	Fixed
GABAB_si_to_si	**0.403**	**0.598**
GABAB_si_to_sp	**0.445**	**0.716**
GABAB_si_to_ss	0.000	Fixed
GABAB_sp_to_sp	**0.652**	**0.805**
GABAB_ss_to_ss	**0.720**	**0.678**
GABAB_tp_to_tp	0.380	Fixed
NMDA_dp_to_di	0.000	Fixed
NMDA_dp_to_tp	0.162	Fixed
NMDA_rl_to_rt	0.000	Fixed
NMDA_rl_to_ss	0.208	Fixed
NMDA_si_to_si	-0.122	Fixed
NMDA_si_to_sp	**0.431**	**0.787**
NMDA_sp_to_dp	0.206	Fixed
NMDA_sp_to_si	0.206	Fixed
NMDA_sp_to_sp	0.341	**0.929**
NMDA_ss_to_rl	0.124	Fixed
NMDA_ss_to_sp	**0.561**	**0.746**
NMDA_tp_to_rl	0.162	Fixed
NMDA_tp_to_ss	0.000	Fixed
TC	**0.753**	**0.699**
scale_NMDA	**0.411**	**0.806**

Table 3: Baseline parameter recovery for the full and reduced model. “Fixed" indicates the parameter was fixed in the reduced model. Parameters with an ICC > 0.4 are in bold.

To further ensure robust parameter recovery assessment, we performed additional outlier removal using a combined approach that identified outliers based on both recovery accuracy (absolute differences between true and estimated values exceeding 1.5 ×

IQR + median or modified Z-score > 3.5) and extreme parameter values (values beyond 3 standard deviations from the mean). This combined outlier detection removed subjects with either poor parameter recovery or biologically implausible parameter values. Table 3 presents the parameter recovery results without outlier removal.

For the two parameters showing significant group differences, we present detailed recovery analysis for both full and reduced models before and after outlier removal (Fig. 10). The reduced model showed substantial improvements in parameter recovery.

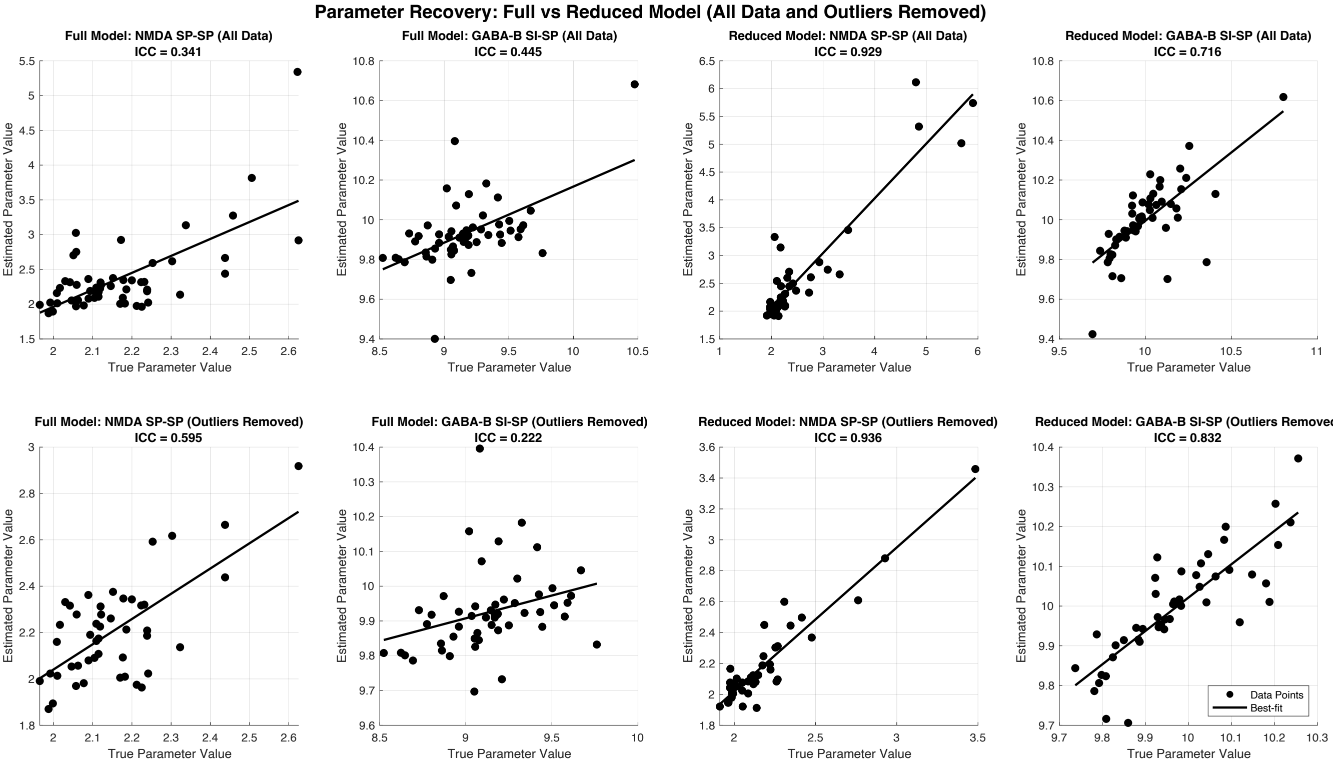

Figure 10: Parameter recovery analysis for NMDA SP-SP and GABA-B SI-SP parameters. Scatter plots show true versus estimated parameter values for both full and reduced models, before and after outlier removal. Each row represents one parameter (top: NMDA SP-SP, bottom: GABA-B SI-SP), with full model results on the left and reduced model results on the right. Black regression lines indicate the best-fit relationship between true and estimated values.

To assess parameter identifiability under degraded signal-to-noise conditions, we systematically reduced the parameter encoding the precision of the data (λ) in the reduced model. For each participant, we generated three synthetic datasets by adjusting the log-precision parameter to achieve +25%, +50%, and +100% increases in residual variance relative to baseline. Based on our fitted models ($R^2 \approx 0.9$), this corresponded to increasing residual variance from 10% to 12.5%, 15%, and 20% of total variance, respectively. The modified parameters were used to generate self-consistent power spectra through the model's transfer function (Tab. 4).

All synthetic datasets were fitted using the reduced model structure. Parameter recovery was quantified using ICC between true and estimated parameters, following the baseline analysis procedure.

Parameter	ICC (25% noise)	ICC (50% noise)	ICC (100% noise)
AMPA_dp_to_di	Fixed	Fixed	Fixed
AMPA_dp_to_tp	**0.767**	**0.722**	0.304
AMPA_rl_to_rt	Fixed	Fixed	Fixed
AMPA_rl_to_ss	**0.848**	**0.802**	**0.672**
AMPA_sp_to_dp	**0.503**	0.300	0.204
AMPA_sp_to_si	**0.730**	**0.538**	0.400
AMPA_ss_to_rl	**0.820**	**0.749**	**0.481**
AMPA_ss_to_sp	**0.735**	**0.554**	0.341
AMPA_tp_to_rl	Fixed	Fixed	Fixed
AMPA_tp_to_ss	Fixed	Fixed	Fixed
CT	**0.838**	**0.703**	**0.596**
GABAA_di_to_di	**0.825**	**0.732**	**0.612**
GABAA_di_to_dp	**0.685**	**0.659**	**0.464**
GABAA_di_to_tp	**0.883**	**0.620**	**0.490**
GABAA_dp_to_dp	**0.566**	0.089	**0.512**
GABAA_rl_to_rl	Fixed	Fixed	Fixed
GABAA_rt_to_rl	**0.620**	**0.454**	0.218
GABAA_rt_to_rt	Fixed	Fixed	Fixed
GABAA_si_to_si	**0.961**	**0.884**	**0.783**
GABAA_si_to_sp	**0.602**	**0.465**	0.342
GABAA_si_to_ss	Fixed	Fixed	Fixed
GABAA_sp_to_sp	**0.772**	**0.627**	**0.424**
GABAA_ss_to_ss	**0.826**	**0.750**	**0.486**
GABAA_tp_to_tp	**0.889**	**0.807**	0.222
GABAB_di_to_di	Fixed	Fixed	Fixed
GABAB_di_to_dp	Fixed	Fixed	Fixed
GABAB_di_to_tp	Fixed	Fixed	Fixed
GABAB_dp_to_dp	Fixed	Fixed	Fixed
GABAB_rl_to_rl	Fixed	Fixed	Fixed
GABAB_rt_to_rl	**0.861**	**0.731**	**0.547**
GABAB_rt_to_rt	Fixed	Fixed	Fixed
GABAB_si_to_si	**0.857**	**0.792**	**0.592**
GABAB_si_to_sp	**0.681**	**0.672**	**0.447**
GABAB_si_to_ss	Fixed	Fixed	Fixed
GABAB_sp_to_sp	**0.877**	**0.863**	**0.716**
GABAB_ss_to_ss	**0.916**	**0.840**	**0.444**
GABAB_tp_to_tp	Fixed	Fixed	Fixed
NMDA_dp_to_di	Fixed	Fixed	Fixed
NMDA_dp_to_tp	Fixed	Fixed	Fixed
NMDA_rl_to_rt	Fixed	Fixed	Fixed
NMDA_rl_to_ss	Fixed	Fixed	Fixed
NMDA_si_to_si	Fixed	Fixed	Fixed
NMDA_si_to_sp	**0.623**	**0.558**	0.155
NMDA_sp_to_dp	Fixed	Fixed	Fixed
NMDA_sp_to_si	Fixed	Fixed	Fixed
NMDA_sp_to_sp	**0.925**	**0.852**	**0.627**
NMDA_ss_to_rl	Fixed	Fixed	Fixed
NMDA_ss_to_sp	**0.674**	0.254	0.120
NMDA_tp_to_rl	Fixed	Fixed	Fixed
NMDA_tp_to_ss	Fixed	Fixed	Fixed
TC	**0.860**	**0.725**	**0.543**
scale_NMDA	**0.711**	**0.631**	**0.530**

Table 4: Parameter recovery under varying noise levels for the reduced model. Parameters with an ICC > 0.4 are in bold.

4.11 Parametric Empirical Bayes

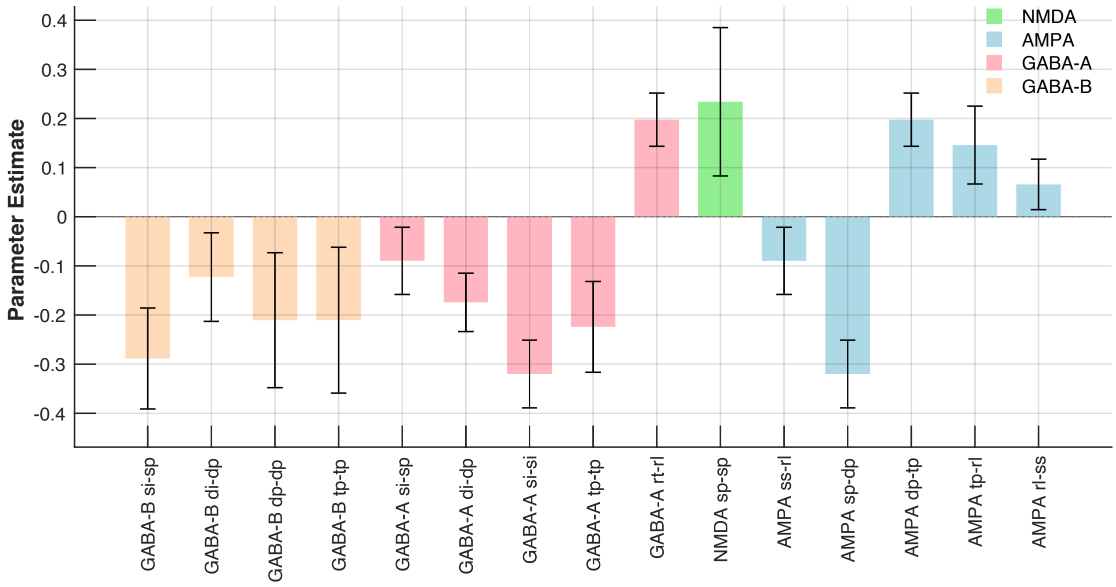

Figure 11: Group differences in DCM parameters between schizophrenia patients and controls identified using Parametric Empirical Bayes analysis (posterior probability > 90%). Bars represent parameter estimates (positive values indicate increases in schizophrenia), and error bars show 90% Bayesian confidence intervals. Parameters are grouped by neurotransmitter type (NMDA: green, AMPA: blue, GABA-A: pink, GABA-B: peach). Connection labels indicate source and target neural populations (ss: spiny stellate cells, sp: superficial pyramidal cells, si: superficial interneurons, dp: deep pyramidal cells, di: deep interneurons, tp: thalamic projection neurons, rl: relay population).

Group differences in DCM parameters were additionally analyzed using Parametric Empirical Bayes (PEB; spm_dcm_peb) 75. PEB implements a hierarchical Bayesian version of a general linear model that assesses parameter differences between groups while accounting for both parameter expectations and their covariances. This is particularly advantageous over classical statistical tests as parameters with higher uncertainty (e.g., from less well-fitting models) contribute proportionally less to the inference. At the first level, individual subject DCMs were combined into a Group DCM (GCM) structure. The second level comprised a design matrix with a constant term (group mean) and a binary regressor coding group membership (controls: -1, schizophrenia: 1).

A key feature of our PEB implementation was the use of Bayesian Model Averaging (BMA)[76] to account for uncertainty in parameter estimates. Rather than selecting a single 'winning' model, BMA provides parameter estimates weighted by the evidence for each model in which they appear. Group differences were identified using a posterior probability threshold of 90%, representing the probability that a parameter differs from zero given the data and model. It's important to note that the 90% threshold used here represents a Bayesian inference threshold rather than a classical statistical significance level.

The analysis revealed several altered connection strengths between schizophrenia patients and controls (figure 11). Notably, we observed increased NMDA-mediated self-connections in superficial pyramidal cells and decreased GABA-B-mediated connections from superficial interneurons, consistent with our primary analysis. The PEB framework also identified additional alterations in AMPA-mediated connections and GABA-A-mediated inhibitory control within specific cortical circuits.

4.12 Parameter Correlations

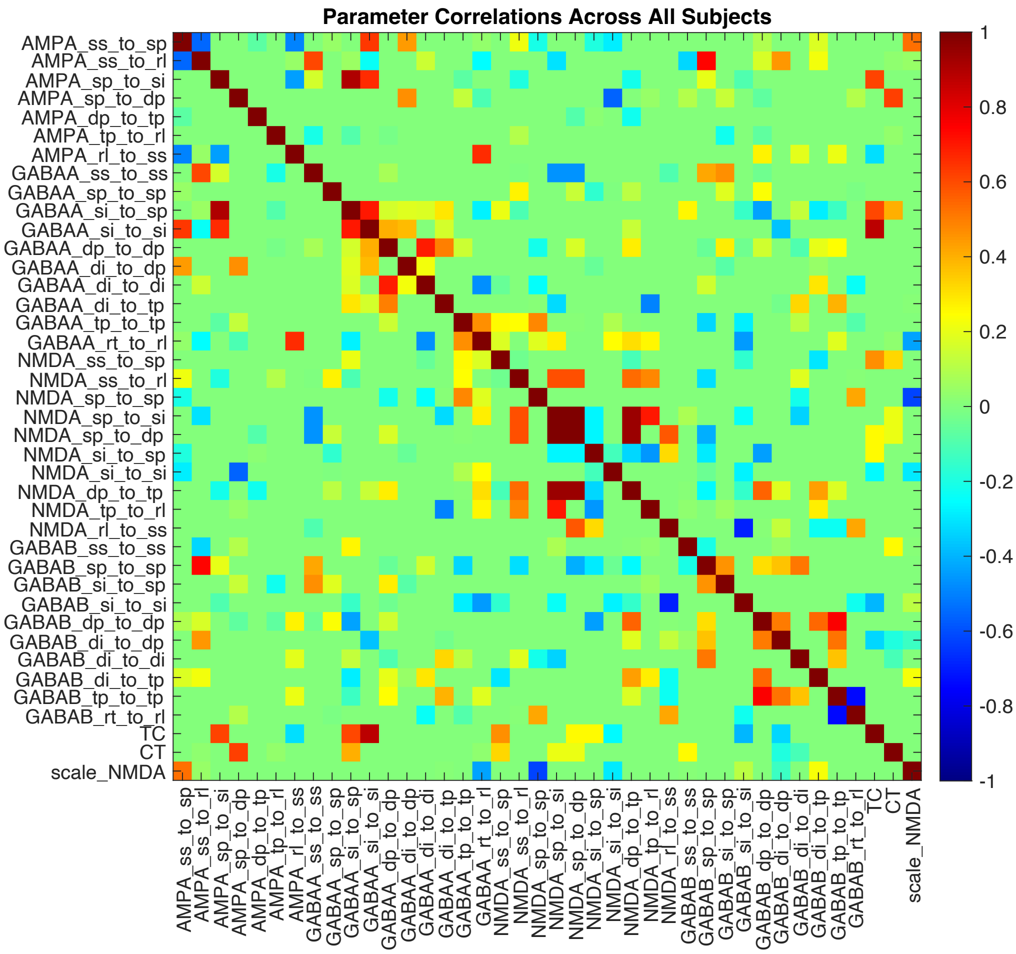

Figure 12: Correlation matrix of DCM parameters across all participants. The matrix shows significant correlations (FDR-corrected p < 0.05) between different connection parameters in the thalamo-cortical model. Parameters include AMPA, GABAA, GABAB, and NMDA receptor-mediated connections between different neural populations. Non-significant correlations are set to zero. The color scale ranges from -1 (dark blue, negative correlation) through 0 (green, no correlation) to +1 (dark red, positive correlation). TC and CT represent thalamo-cortical and cortico-thalamic connections respectively, while scale_NMDA represents the NMDA receptor scaling parameter.

To better understand the relationships between different synaptic mechanisms in our model, we performed correlation analyses across all model parameters. This analysis helps reveal how different synaptic connections might interact or compensate for each other within the thalamo-cortical circuit.

We calculated correlation coefficients between all pairs of parameters and applied FDR correction to control for multiple comparisons. Only correlations that remained significant after FDR correction ($p_{FDR} < 0.05$) were considered. This conservative approach helps identify the most robust relationships between parameters while minimizing false positives.

4.13 Parameter Contribution Analysis

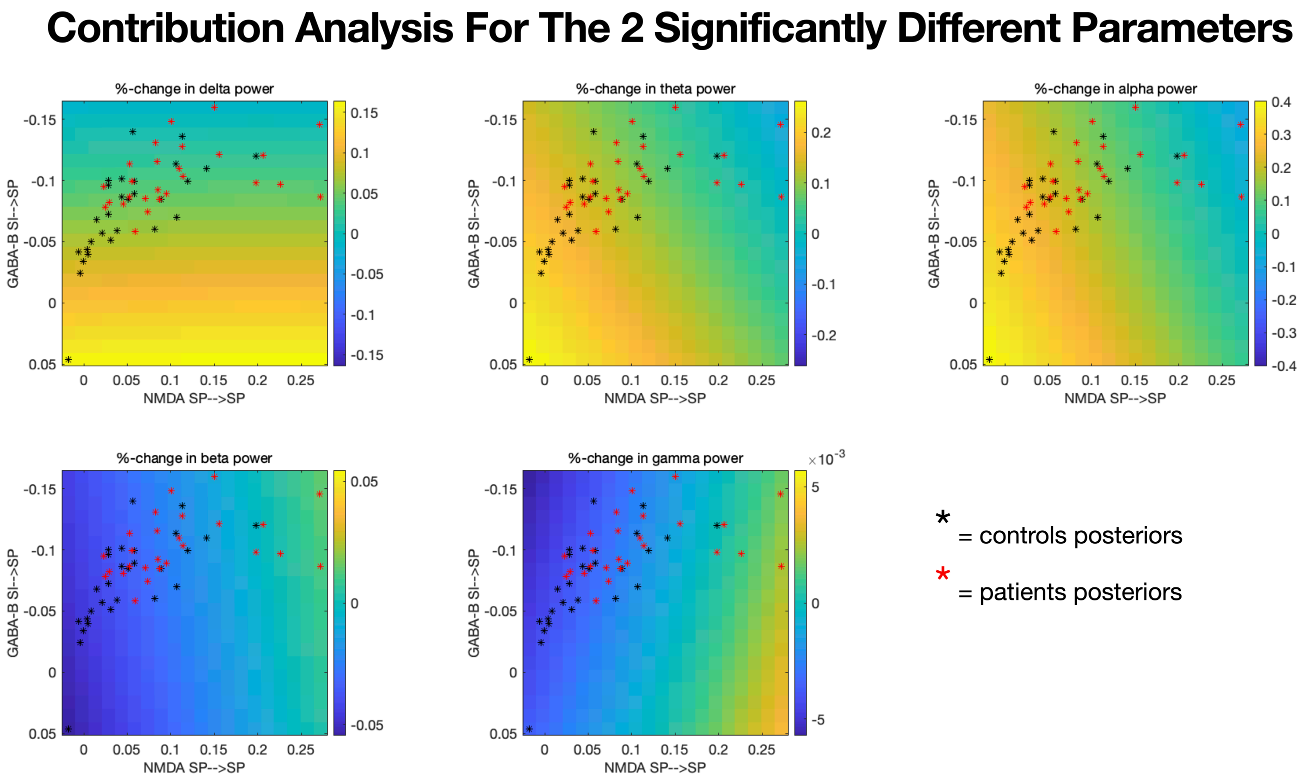

Figure 13: ***Contribution analysis.*** Contribution analysis for the two significantly different parameters: NMDA-mediated self-connections within superficial pyramidal (SP) cells and GABA-B-mediated connections from superficial interneurons (SI) to SP cells. The heatmaps represent the contribution of these parameters to the percent changes in power, with color indicating the magnitude and direction of the contribution. Black stars represent posterior distributions for controls, while red stars represent posteriors for patients. The axes show the range of parameter values explored. The parameter ranges were determined by the minimum and maximum values observed across all participants. While the analysis highlights potential interactions between these parameters, the findings remain exploratory.

To better understand how our two significantly different parameters contribute to the model's spectral output, we performed a focused contribution analysis. Specifically, we examined how NMDA-mediated recurrent excitation among superficial pyramidal cells and $GABA_B$-mediated inhibition from superficial interneurons to superficial pyramidal cells influence the power spectrum. This targeted analysis provides mechanistic insight into how these specific synaptic alterations might generate the spectral differences observed between groups.

Importantly, the contribution analysis revealed an apparent paradox: the individual effects of these statistically different parameters on spectral features (changes of 0.4% in alpha and 0.005% in gamma power) were substantially smaller than the observed group differences (10-15%). Moreover, their isolated effects sometimes showed opposite patterns to the observed group differences (especially in alpha and gamma). This finding demonstrates that statistical differences in parameters should not be interpreted as causal explanations for the spectral abnormalities: identifying statistical differences in circuit parameters does not necessarily reveal which parameters are mechanistically responsible for altered circuit function. Computational simulations,

however, can reveal how parameters interact within the circuit to produce the observed spectral alterations.

4.14 Step-wise regression & negative symptoms

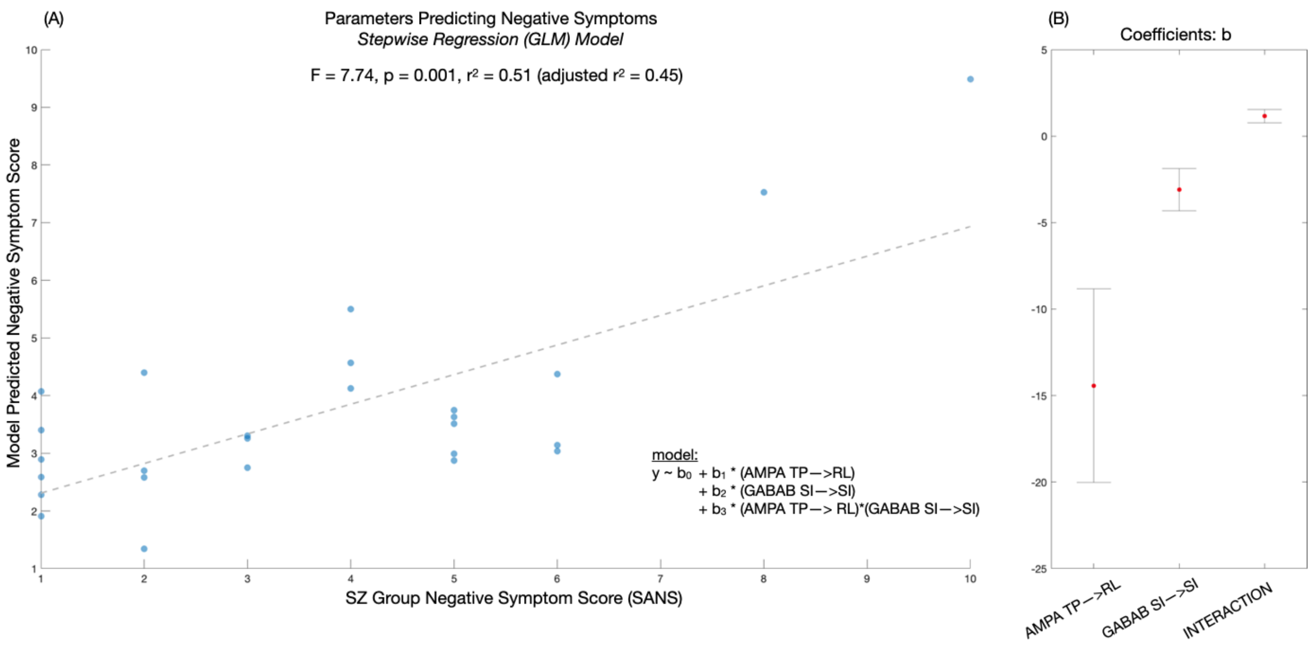

Figure 14: ***Stepwise regression model predicting negative symptoms in schizophrenia.*** **(A)** Scatter plot comparing observed negative symptom scores (SANS) with predicted scores from the stepwise regression model. Each point represents an individual with schizophrenia. The dashed line shows the model fit (F = 7.74, p = 0.001, r^2 = 0.51, adjusted r^2 = 0.45). The model equation includes synaptic parameters: AMPA (TP→RL), GABAB (SI→SI), and their interaction. **(B)** Coefficient plot showing the relative contributions (beta weights) of each predictor in the model. Error bars indicate 95% confidence intervals.

Our study extended beyond identifying group differences in model parameters to examining their relationship with clinical symptoms. Without strong prior hypotheses regarding neurobiological mechanisms underwriting negative symptoms - and acknowledging the "multi-system" nature of psychiatric physiology, we employed data-driven, stepwise regression to determine which parameters were significantly correlated to negative symptoms in individuals with schizophrenia. Stepwise regression has known drawbacks, including potential overfitting, sensitivity to collinearity between predictors, and a tendency to generate false positives[77]. More modern approaches might offer theoretical advantages in parameter selection. However, given our relatively small sample size compared to the number of parameters, any regression-based approach would face inherent limitations. We therefore present these results as exploratory findings that require validation in larger samples using more robust statistical methods.

The stepwise regression systematically evaluated combinations of model parameters, iteratively including or excluding them based on their association strength with negative symptom severity. This data-driven approach allows for an exploration of the links between neurophysiological parameters and severity of symptoms. We here foucs only on negative symptoms since the individuals with schizophrenia did not show any acute psychotic symptoms. The final model achieved statistical significance (F = 7.74, p = 0.001) and explained a substantial portion of the variance in negative symptom scores (r^2 = 0.51, adjusted r^2 = 0.45).

The model identified three key parameters as predictors of negative symptom severity: AMPA-mediated connectivity from thalamic projection (TP) neurons to relay (RL) neurons (AMPA TP-RL), GABA-B-mediated self-inhibition of superficial interneurons ($GABA_B$ SI-SI), and an interaction term between AMPA TP-RL and $GABA_B$ SI-SI. The analysis revealed a positive correlation between predicted and observed negative symptom scores, indicating that the model generally captures the trend in symptom severity. The AMPA TP-RL parameter showed a strong negative association with symptom severity (b = -14.97), while the $GABA_B$ SI-SI parameter demonstrated a a less pronounced negative association (b = -3.25). The interaction term between these two parameters showed a positive relationship with symptom severity (b = 1.99).

These findings suggest that alterations in thalamocortical AMPA signaling and local GABA-B-mediated inhibition, as well as their interaction, may play significant roles in the manifestation of negative symptoms in schizophrenia.

The involvement of thalamic projection neurons in predicting negative symptom severity is particularly interesting. Layer 6 cortical neurons projecting back to the thalamus play a critical role in modulating thalamic output and, subsequently, thalamo-cortical feedback[78]. Reduced efficacy in these projections could lead to diminished capacity to modulate and synchronize thalamo-cortical activity, potentially contributing to the cognitive and motivational deficits characteristic of negative symptoms[79]. The decreased modulation from layer 6 cortical neurons to the thalamus, coupled with altered inhibitory control from superficial interneurons, could lead to a dysfunctional thalamo-cortical loop. In this scenario, both the input to and output from the thalamus are not adequately regulated, potentially underlying the more pronounced negative symptoms seen in schizophrenia.

Our findings align with and extend the theory of thalamo-cortical dysfunction in schizophrenia. This hypothesis states that abnormal interactions between the thalamus and cortex contribute to the cognitive, perceptual, and emotional disturbances seen in schizophrenia[80,81]. Specifically, it suggests that disrupted filtering and integration of sensory information by the thalamus leads to aberrant cortical processing and, consequently, to various symptoms of the disorder. This theory has been supported by a number of neuroimaging, electrophysiological, and post-mortem studies[11,82,83].

4.15 Principal Component Analysis Loadings

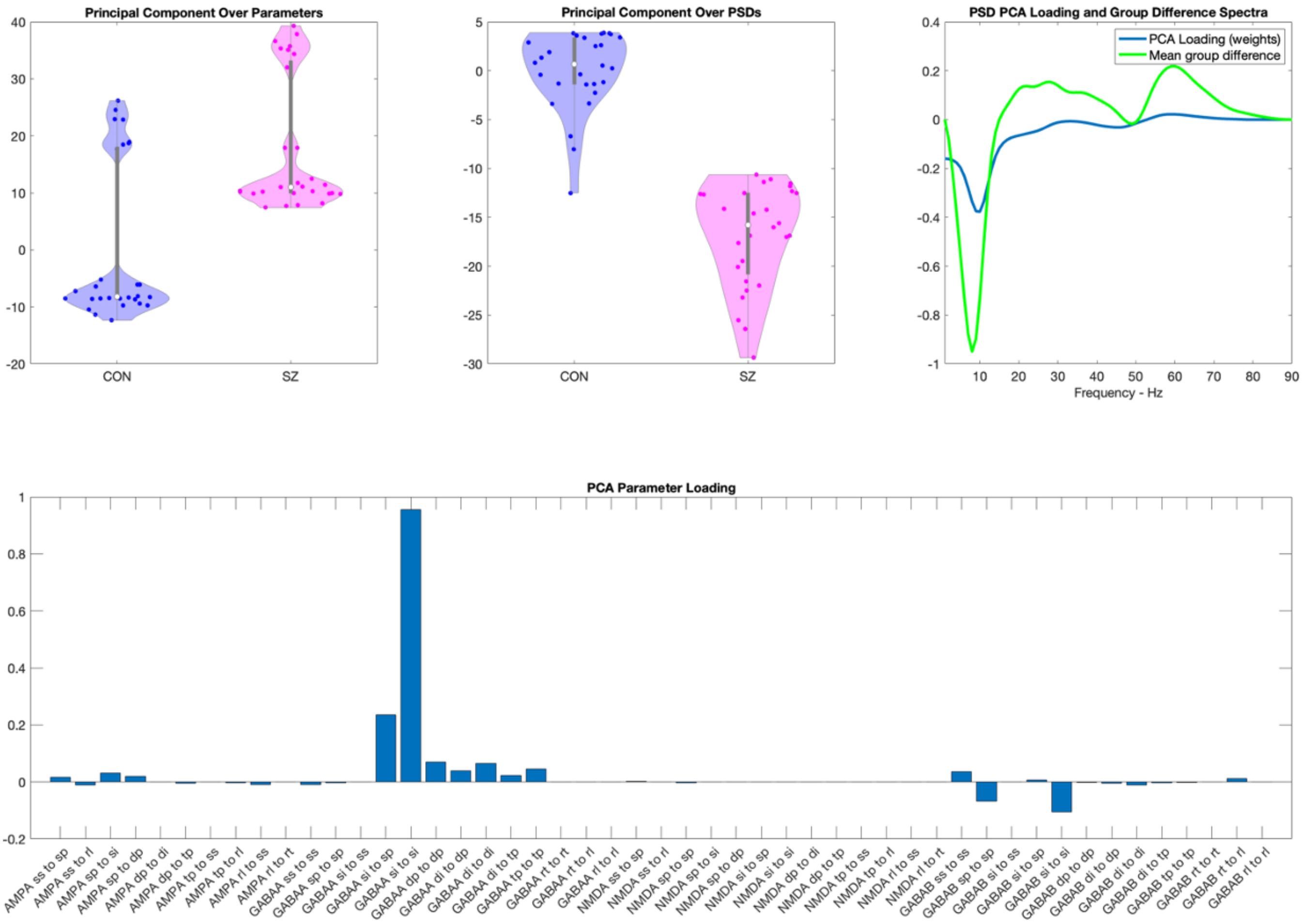

Figure 15: ***Parameter Contributions and Subgroup Differentiation via PCA.*** PCA loadings and subgroup differentiation based on parameter contributions. The top row shows violin plots of the PCA scores for control (CON) and schizophrenia (SZ) groups, highlighting the formation of two distinct subgroups within each group. The third panel illustrates the PCA loading weights across frequencies (blue line) compared to the mean group differences (green line). The bottom bar plot represents the contribution of individual parameters to the PCA loadings, with specific parameters dominating the separation.
